# A biodegradable antibiotic delivery system based on poly-(trimethylene carbonate) for the treatment of osteomyelitis

**DOI:** 10.3109/17453670903350040

**Published:** 2009-10-01

**Authors:** Daniëlle Neut, Otto S Kluin, Bart J Crielaard, Henny C van der Mei, Henk J Busscher, Dirk W Grijpma

**Affiliations:** ^1^Department of Biomedical Engineering, University Medical Center Groningen and University of GroningenGroningenthe Netherlands; ^2^Department of Orthopaedic Surgery, University Medical Center Groningen and University of GroningenGroningenthe Netherlands; ^3^Institute of Biomedical Technology (BMTI) and Department of Polymer Chemistry and Biomaterials, University of TwenteEnschedethe Netherlands

## Abstract

**Background and purpose** Many investigations on biodegradable materials acting as an antibiotic carrier for local drug delivery are based on poly(lactide). However, the use of poly(lactide) implants in bone has been disputed because of poor bone regeneration at the site of implantation. Poly(trimethylene carbonate) (PTMC) is an enzymatically degradable polymer that does not produce acidic degradation products. We explored the suitability of PTMC as an antibiotic releasing polymer for the local treatment of osteomyelitis.

**Methods** This study addressed 2 separate attributes of PTMC: (1) the release kinetics of gentamicin-loaded PTMC and (2) its behavior in inhibiting biofilm formation. Both of these characteristics were compared with those of commercially available gentamicin-loaded poly(methylmethacrylate) (PMMA) beads, which are commonly used in the local treatment of osteomyelitis.

**Results** In a lipase solution that mimics the in vivo situation, PTMC discs with gentamicin incorporated were degraded by surface erosion and released 60% of the gentamicin within 14 days. This is similar to the gentamicin release from clinically used PMMA beads. Moreover, biofilm formation by *Staphylococcus aureus* was inhibited by approximately 80% over at least 14 days in the presence of gentamicin-loaded PTMC discs. This is similar to the effect of gentamicin-loaded PMMA beads. In the absence of the lipase, surface erosion of PTMC discs did not occur and gentamicin release and biofilm inhibition were limited.

**Interpretation** Since gentamicin-loaded PTMC discs show antibiotic release characteristics and biofilm inhibition characteristics similar to those of gentamicin-loaded PMMA beads, PTMC appears to be a promising biodegradable carrier in the local treatment of osteomyelitis.

## Introduction

Osteomyelitis is characterized by progressive inflammatory bone destruction (Mader et al. 1997b). In general, chronic osteomyelitis cannot be eradicated solely by intravenous administration of antibiotics (Haleem et al. 2004). As the infected necrotic focus within the bone is often surrounded by sclerotic avascular bone, it is almost unreachable using systemic antibiotics. Moreover, the necrotic bone forms a suitable substrate for biofilm formation, which reduces the antibiotic sensitivity of the infecting pathogens and makes the infection difficult to treat (Gristina et al. 1985, Stewart and Costerton 2001). Thus, in the management of chronic osteomyelitis, it is generally necessary to remove the infected bone and tissue followed by implantation of an antibiotic delivery system that can provide high local antibiotic concentrations for extended periods of time.

Sustained high local antibiotic concentrations are currently achieved by the implantation of gentamicin-loaded poly(methylmethacrylate) (PMMA) beads. Although these beads have been used to treat osteomyelitis for many years (Buchholz et al. 1984, Klemm 2001), their major drawback is the additional surgery required to remove the beads after therapy, as PMMA is not biodegradable. Also, PMMA beads do not release all of their gentamicin content: after 2 weeks in situ, only 20–70% of the total amount of gentamicin incorporated in the beads is released and the gentamicin concentrations in serum drop considerably thereafter (Walenkamp et al. 1986). Continuous, high release rates for prolonged periods of time are necessary, however, as the polymer bead surface itself may provide an ideal surface for biofilm formation that inhibits diffusion of antibiotics, leading to recurrent infection (Mader et al. 1999, Kanellakopoulou and Giamarellos-Bourboulis 2000, Neut et al. 2001, 2003). Incomplete drug release, possible colonization of PMMA beads by bacteria (Neut et al. 2001, 2003), and the requirement of surgical removal of these beads have prompted investigations on the use of biodegradable materials as antibiotic carriers for local delivery, especially for the treatment of osteomyelitis with small volumes of dead space.

Currently, the biodegradable carrier materials most often investigated have been based on poly(lactide) (PLA) and/or poly(glycolide) (PGA) (Lin et al. 1999, Garvin and Feschuk 2005, Koort et al. 2006, Naraharisetti et al. 2006). Biodegradable carriers can sustain high local antibiotic concentrations for up to 8 months, whereas antibiotic release from PMMA beads can diminish to values below the detection limit within 12 days (Mader et al. 1997a). Furthermore, biodegradable carriers can release all of their antibiotic content (Koort et al. 2006).

The use of PLA and PGA implants has been disputed (Barber and Dockery 2006, Bergsma et al. 1995, Böstman 1991, Böstman et al. 2005). An important issue relates to the biocompatibility of these polymers in relation to human bone. There have been several studies reporting an inflammatory foreign-body reaction, reduced bone regeneration, or increased bone resorption at the site of implantation when PLA or PGA implants were used (Böstman et al. 1990, 2005, Böstman 1991, Gunatillake and Adhikari 2003). It has been suggested that acidic degradation products and a subsequent decrease in local pH are the cause of decreased bone regeneration (Gunatillake and Adhikari 2003). Thus, PLA and/or PGA do not appear to be the most suitable carriers for use in bone, and further investigation of alternative materials is required.

One possible candidate material is poly(trimethylene carbonate) (PTMC), a biodegradable polymer that is relatively new in biomedical applications.

In contrast to PLA/PGA, the degradation products of PTMC are not acidic (Zhang et al. 2006). Interestingly, the degradation rate of PTMC in vitro at pH 7 and 37°C appears to be quite low (Zhu et al. 1991), while the degradation rate of PTMC is much faster in vivo (Zhu et al. 1991, Pego et al. 2003, Zhang et al. 2006). The difference between in vitro and in vivo degradation rates suggests that enzymes are involved. Indeed, enzymatic degradation of PTMC and TMC co-polymers has been shown to occur in vitro (Matsumura et al. 2001, Tsutsumi et al. 2002, Zhang et al. 2006). Overall, PTMC has good biocompatibility and degrades enzymatically in a uniform manner by surface erosion; thus, it may provide sustained high antibiotic release rates. This has not been established experimentally.

The objective of this in vitro study was to explore the suitability of PTMC as a biodegradable, antibiotic-releasing carrier, and to compare it with commercially available gentamicin-loaded PMMA beads. The study investigated 2 related issues regarding gentamicin-loaded PTMC: (1) the release kinetics and (2) its ability to inhibit biofilm formation.

## Material and methods

### Materials

Polymer-grade 1,3-trimethylene carbonate (TMC) was purchased from Boehringer Ingelheim (Ingelheim, Germany). Gentamicin sulfate was purchased from Sigma-Aldrich (St. Louis, MO), and analytical-grade chloroform and diethyl ether were purchased from Merck BV (Schiphol-Rijk, the Netherlands). A lipase solution from Thermomyces lanuginosus (EC 3.1.1.3., minimum 105 units/g) was purchased from Sigma-Aldrich and used as received. Antibiotic-loaded poly(methylmethacrylate) (PMMA) beads are commercially available under the name Septopal (Biomet Europe, Darmstadt, Germany). Each Septopal bead (diameter: 7 mm) contains 7.5 mg of gentamicin sulfate (corresponding to 4.5 mg of gentamicin base). Staphylococcus aureus 0734 (MIC-value of gentamicin amounts to 0.50 μg/mL) is one of the most commonly involved bacterial strains in osteomyelitis (Lew and Waldvogel 2004) and was retrieved from an infected prosthesis.

### Preparation of gentamicin-loaded PTMC

High-molecular-weight PTMC (Mn = 388 × 103 g/mol) was synthesized, purified, and characterized as previously described (Zhang et al. 2006). 2 solutions of PTMC in chloroform were prepared (4% w/v). Gentamicin sulfate (10% w/w with respect to the polymer) was added to one of the solutions and mixed for 5 min using an ultrasonic bath, followed by extensive stirring for 24 h. This suspension was then dropped into an excess of diethyl ether. As PTMC and gentamicin sulfate do not dissolve in diethyl ether, this induces precipitation of the polymer—through which gentamicin is homogeneously distributed. The second solution of PTMC in chloroform was also precipitated in diethyl ether, but it did not contain gentamicin sulfate.

PTMC precipitates with and without gentamicin were compression-molded at 50°C into films of 500 μm thickness using a laboratory press. Finally, discs with a diameter of 5 mm were punched out of the films.

### Scanning electron microscopy (SEM)

The surface erosion of gentamicin-loaded PTMC was visualized using SEM. A series of SEM micrographs of PTMC discs were taken after immersion in phosphate-buffered saline (PBS), pH 7.0, or in the lipase solution. After 3, 7, and 10 days, discs were taken out of the solutions for SEM evaluation. Discs were sputter-coated with gold/palladium and examined at 2.0 kV in a JEOL field emission scanning electron microscope type 6301F.

### Release of gentamicin

Gentamicin release was determined at 37°C in PBS (in the absence of lipase) and in a lipase solution, which induces surface erosion of PTMC (Zhang et al. 2006). 3 gentamicin-loaded PTMC discs were each immersed in 10 mL PBS or lipase solution and incubated at 37°C for 2 weeks. Liquid samples (500 μL) from both solutions were taken at 6, 24, 48, 72, 168, and 336 h and stored at 4°C before analysis. For comparison, the gentamicin release from gentamicin-loaded Septopal PMMA beads was measured in PBS (n = 3).

Gentamicin concentrations were measured using a procedure described by Zhang et al. (1994). Briefly, an o-phtaldialdehyde reagent was made and stored for 24 h in a dark environment. A gentamicin-containing aliquot, o-phtaldialdehyde reagent, and isopropanol were mixed in equal proportions and stored for 30 min at room temperature. Upon reaction of o-phtaldialdehyde with the amino groups of gentamicin, chromophoric products were obtained. Their absorbances were measured at 332 nm using a Spectronic 20 Genesys spectrophotometer (Spectronic Instruments, Inc. Rochester, NY). After measuring the absorbances of a series of solutions of known gentamicin concentrations to prepare a calibration curve, the absorbance could be related to the gentamicin concentration of each aliquot.

Gentamicin concentrations in lipase solutions could not be determined spectrophotometrically due to interference of the enzyme. An indirect bacterial inhibition assay, described and validated by Joosten et al. (2005), was therefore used to determine gentamicin concentrations in the lipase samples. Tryptone soya broth (TSB; Oxoid, Basingstoke, UK) agar plates were smeared with the S. aureus strain. From each lipase sample and gentamicin solution of known concentration, 15 μL was dropped onto a TSB agar plate; the plates were then incubated at 37°C for 24 h. The antimicrobial agent in the samples inhibited bacterial growth during incubation, which resulted in zones of inhibition. The areas of the inhibition zones were measured after incubation, from which the gentamicin concentration was deduced by comparison with the inhibition zones corresponding to the known gentamicin concentrations.

### Inhibition of biofilm formation

The extent to which the released amount of antibiotics inhibits the formation of a biofilm is an index of the efficacy of the antibiotic-loaded carrier. To study biofilm formation, 2 types of bacterial growth media were prepared: TSB and a 4:1 mixture of TSB and lipase solution to allow enzymatic degradation of PTMC. 3 non-loaded PTMC discs and 3 gentamicin-loaded PTMC discs were immersed in 10 mL TSB or 10 mL TSB/lipase medium for 2 weeks at 37°C. In addition, 3 gentamicin-loaded PMMA beads were immersed in 10 mL of TSB. Medium was collected and replaced with new medium after 24, 48, 72, 144, 168, 312, and 336 h to obtain samples containing the antibiotic released during the first, second, third, seventh, and fourteenth day.

These elution media were used for the formation of *S. aureus* biofilms in 96-well plates (Nunc-Immuno 96 microwell plates; Thermo Fisher Scientific) under stationary conditions. 2 μL of bacterial inoculum (approximately 1 × 10^6^ CFU/μL) was added to wells containing 198 μL of TSB. After 24 h of incubation at 37°C, the medium was removed and the plates were washed 3 times with 220 μL of PBS. Subsequently, each well was stained with 200 μL of 1% (w/v) crystal violet in water for 30 min. After staining, the plates were gently rinsed 3 times with demineralized water. Quantitative analysis of biofilm formation was performed by adding 200 μL of ethanol/acetone (80:20) to solubilize the crystal violet. The absorbance of this crystal violet solution was measured using a FLUOstar Optima plate reader (BMG Labtech GmbH, Offenburg, Germany) at a wavelength of 575 nm. The absorbance (A) is proportional to the amount of crystal violet, which is directly proportional to the amount of biofilm grown in each well.

The maximal amount of biofilm formation was defined as the amount grown in elution media collected from non-loaded PTMC discs, and the percentage of biofilm inhibition was calculated according to the following equation:

**Figure:**
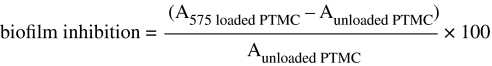


For gentamicin-loaded PMMA beads, the maximal amount of biofilm formation was defined as the amount grown in freshly inoculated TSB medium, as non-loaded PMMA beads are not commercially available. All experiments included 6 replicate wells and were performed in triplicate with separately cultured bacteria.

### Statistics

In evaluating gentamicin release characteristics and inhibition of biofilm formation, Student's t-test for independent samples was used. A 95% (p < 0.05, two-tailed) confidence interval was applied for statistical significance.

## Results

### Scanning electron microscopy

**Figure 1.**
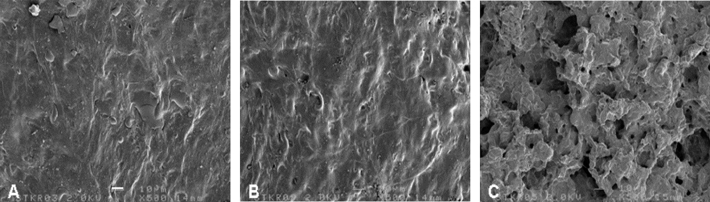
Scanning electron micrographs of the surface of PTMC discs prior to immersion (A), after immersion in PBS for 7 days (B), and after immersion in lipase solution for 7 days (C). The bar represents 10 μm.

The surface topographies of PTMC immersed for 3, 7, or 10 days in PBS and in lipase solution were visualized with SEM. Examples of micrographs after immersion for 7 days are shown in Figure 1. In PBS, PTMC showed no signs of degradation (Figure 1B), as the surface topography looked exactly the same as prior to immersion (Figure 1A) irrespective of the number of days the specimens were immersed. In contrast, PMTC in lipase solution clearly showed surface erosion of the polymer after 7 days, (Figure 1C). This was also observed after 3 or 10 days of immersion (micrographs not shown).

### Gentamicin release

**Figure 2.**
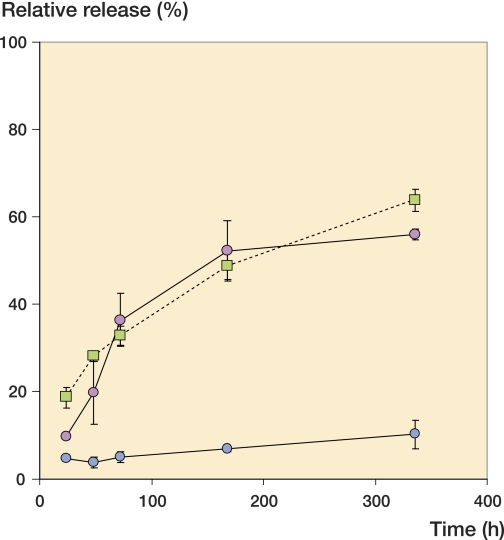
Cumulative gentamicin release from PTMC discs in lipase solutions (red circles) and from PTMC discs (blue circles) and Septopal beads (green squares) in PBS as a function of time. The amount of gentamicin released is expressed as a percentage of the amount of gentamicin incorporated per disc or bead. The error bars indicate the standard deviation of 3 experiments performed in each group.

The cumulative release of gentamicin from PTMC discs in PBS, PTMC discs in lipase solution, and PMMA beads in PBS is shown graphically in Figure 2 and numerically in the Appendix (see supplementary data), together with detailed statistical analyses. PTMC in PBS released about 10% of its total load within 2 weeks, while PMMA released about 60%. The release of gentamicin from PTMC in lipase was more rapid than the release of gentamicin from PTMC in PBS (p < 0.05). In lipase solution, PTMC discs released approximately 60% of their total load within 2 weeks. The rate of gentamicin release from PTMC with a degrading surface was similar to that of the gentamicin-loaded PMMA beads.

### Inhibition of biofilm formation

**Figure 3.**
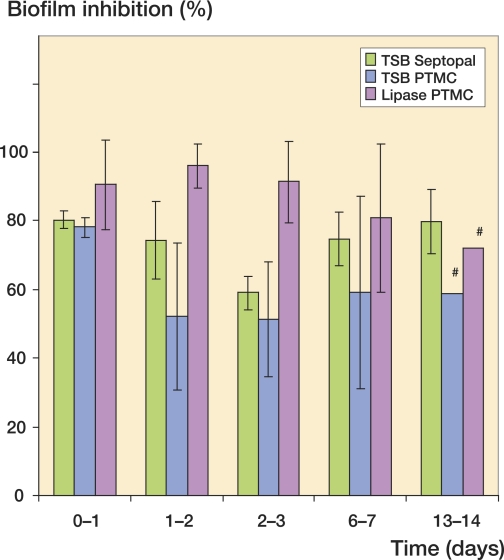
Inhibition of S. aureus biofilm formation after 24-h release of gentamicin in TSB or TSB/lipase medium by gentamicin-loaded PTMC discs and Septopal beads after 1, 2, 3, 7, and 14 days. The extent of inhibition is expressed relative to non-loaded PTMC (100%). The bars represent the means of 3 separate experiments, with the standard deviation shown by the error bars.

The biofilm inhibition after 24 h in growth medium containing gentamicin released from gentamicin-loaded PTMC discs and PMMA beads during the first, second, third, seventh, and fourteenth day is graphically presented in Figure 3 and numerically in the Appendix (see supplementary data), together with detailed statistical analyses. During the first day of release in growth medium, similar inhibition by PTMC discs and PMMA beads was seen (approximately 80%). However, after the first day of release, PTMC in growth medium with no lipase showed less biofilm inhibition than that obtained with PMMA beads.

The inhibition obtained from gentamicin-loaded PTMC discs incubated in lipase-containing growth medium was, however, better than that obtained with similar discs incubated in growth medium only (p < 0.05). After surface erosion in the lipase enzyme solution, gentamicin-loaded PTMC showed continuous, effective inhibition of biofilm formation (Figure 3) similar to that obtained with the PMMA beads.

## Discussion

Gentamicin-loaded PMMA beads constitute an effective drug delivery system for local antibiotic therapy in bone infections. However, after high initial gentamicin concentrations at the site of the infection, the gentamicin concentrations drop considerably (Walenkamp et al. 1986). The main efficacy of gentamicin is therefore immediately after implantation of the beads, and long-term implantation of the beads is of no value in combatting infection. One of the drawbacks of the use of non-biodegradable vehicles for local antibiotic delivery (such as PMMA beads) is the possible bacterial colonization of their surfaces, and biofilm formation. In this respect, biodegradable polymers as carriers of antibiotics that degrade by surface erosion may be preferred, as they do not show long-term release of sub-inhibitory antibiotic concentrations. Moreover, a biofilm is perhaps not as easily formed on a material whose surface is undergoing erosion, as the adhering organisms are shed continuously along with the eroding material.

PTMC is such a surface-eroding polymer. It is an aliphatic polycarbonate, and upon degradation of the carbonate linkages by hydrolysis, carbon dioxide and 1,3-propanediol will be formed. Thus, PTMC degrades without the formation of acidic degradation products and has good compatibility with bone tissue. Subcutaneous implantation of PTMC discs in rats showed a mild-to-moderate tissue reaction around the implant (Pego et al. 2003). Within 1 year, the polymer was completely resorbed and the tissue at the site of implantation had regenerated. Implantation of PTMC rods into the femur and tibia of rabbits gave similar results (Zhang et al. 2006).

PTMC degrades very slowly by hydrolysis in vitro: the polymer is stable in water and in buffered solutions (Zhang et al. 2006). However, in vivo degradation of PTMC subcutaneously implanted in the back of rats was found to be strikingly rapid. The mass of 600-μm thick PTMC samples decreased linearly with time and resorption was nearly complete in 3 weeks (Pego et al. 2003). The difference between the in vitro and in vivo degradation behavior suggests that enzymes are involved in the process, but the specific enzymes involved still have to be identified. Zhang et al. (2006) were able to mimic the in vivo enzymatic degradation of PTMC using lipase solutions.

Interestingly, the degradation of PTMC showed characteristics of a surface erosion process—as the loss of mass could be correlated to the decrease in thickness of the specimen, and the decrease in molecular weight was limited (Zhu et al. 1991, Pego et al. 2003, Zhang et al. 2006). This indicates that contrary to the bulk degradation process observed in the case of PLA and PGA, the degradation of PTMC mainly takes place at the surface of the specimen. In drug delivery applications, a surface-eroding polymer may have advantages over a bulk eroding polymer, as surface erosion allows constant and controllable drug release rates and the drug is not exposed to the environment until the moment it is released.

A crucial point in the treatment of chronic osteomyelitis is the handling of the dead space and gentamicin-loaded PMMA beads can be used to treat the dead space problem. In addition to providing local delivery of antibiotics, these beads will fill the dead space. After the infection is cured, the beads can be removed and the dead space can be filled either with a bone graft or a muscle flap. A specific dead space problem is related to two-stage revision of infected joint prostheses. In these cases, PMMA beads have been used to fill the dead space, i.e. to facilitate the implantation of a new prosthesis. In the examples above, the surgeon uses PMMA beads as non-biodegradable carriers of antibiotics. Accordingly, the use of biodegradable carriers is confined to cases where the dead space problem is considered to be small.

The aim of our study was to explore the suitability of biodegradable antibiotic-loaded PTMC discs in the local treatment of osteomyelitis, especially for cases where the volume of dead space is low. In buffer, PTMC released only small amounts of antibiotics during the first 2 weeks, but in a lipase solution (where PTMC degrades by surface erosion), this was accompanied by a high rate of release of antibiotics similar to that observed for non-biodegradable Septopal beads made from PMMA. In line with this, gentamicin-loaded PTMC showed less inhibition of biofilm formation for 2 weeks under non-surface eroding conditions in buffer than under surface-eroding conditions in lipase solution. Moreover, the inhibitory effect of gentamicin-loaded PTMC in the lipase enzyme solution was similar to that of clinically used Septopal beads. This indicates that under conditions that favor surface degradation of the PTMC discs, the polymer could be a suitable biodegradable carrier of antibiotics in the treatment of osteomyelitis. It should be emphasized, however, that a strict comparison of the antibiotic release behavior of Septopal beads and of our PTMC specimens is not possible due to their different geometries, masses, and degrees of gentamicin loading.

In this study, macroscopic examination showed that the PTMC discs were completely resorbed within 2 weeks upon immersion in the lipase solution. As PTMC degrades by a surface erosion process, it is possible to extend the degradation time by increasing the thickness of the specimens and by reducing the surface-to-volume ratio. It has also been shown that reducing the initial molecular weight of PTMC polymer has the effect of reducing the rate of degradation (Zhang et al. 2006). After implantation for 8 weeks in the femur and tibia of rabbits, the mass loss of high molecular weight PTMC457 rods (60 wt%) was 3 times higher than that of intermediate molecular weight PTMC89 rods (20 wt%). Thus, the antibiotic release characteristics of gentamicin-loaded PTMC can be readily fine-tuned to meet clinical demands.

A next step would be to initiate animal experiments to confirm the release characteristics and antimicrobial efficacy of gentamicin-loaded PTMC discs as seen in this study. Animal experiments should also be carried out to study the long-term effects of gentamicin-loaded PTMC implants on the regeneration of bone. This is necessary to ensure that PTMC is safe during its degradation in bone, and that it can be used as a drug delivery device in orthopedic applications.

In summary, gentamicin-loaded PTMC discs degrading in lipase solution showed antibiotic release kinetics and biofilm inhibition properties that are comparable to those of non-biodegradable gentamicin-loaded PMMA beads. The use of antibiotic-loaded PTMC discs does not require a second surgery for the removal of the beads after therapy; therefore, PTMC-based materials appear to be promising alternative antibiotic carriers for the local treatment of osteomyelitis.
